# Semantic Expectation Effects on Object Detection: Using Figure Assignment to Elucidate Mechanisms

**DOI:** 10.3390/vision6010019

**Published:** 2022-03-21

**Authors:** Rachel M. Skocypec, Mary A. Peterson

**Affiliations:** 1 Visual Perception Lab, Department of Psychology, School of Mind, Brain and Behavior, University of Arizona, Tucson, AZ 85721, USA; 2 Cognitive Science Program, School of Mind, Brain and Behavior, University of Arizona, Tucson, AZ 85721, USA

**Keywords:** object detection, figure assignment, semantic conflict, semantics, semantic network, superordinate-level category

## Abstract

Recent evidence suggesting that object detection is improved following valid rather than invalid labels implies that semantics influence object detection. It is not clear, however, whether the results index object detection or feature detection. Further, because control conditions were absent and labels and objects were repeated multiple times, the mechanisms are unknown. We assessed object detection via figure assignment, whereby objects are segmented from backgrounds. Masked bipartite displays depicting a portion of a mono-oriented object (a familiar configuration) on one side of a central border were shown once only for 90 or 100 ms. Familiar configuration is a figural prior. Accurate detection was indexed by reports of an object on the familiar configuration side of the border. Compared to control experiments without labels, valid labels improved accuracy and reduced response times (RTs) more for upright than inverted objects (Studies 1 and 2). Invalid labels denoting different superordinate-level objects (DSC; Study 1) or same superordinate-level objects (SSC; Study 2) reduced accuracy for upright displays only. Orientation dependency indicates that effects are mediated by activated object representations rather than features which are invariant over orientation. Following invalid SSC labels (Study 2), accurate detection RTs were longer than control for both orientations, implicating conflict between semantic representations that had to be resolved before object detection. These results demonstrate that object detection is not just affected by semantics, it entails semantics.

## 1. Introduction

The question of whether visual perception is influenced by meaning has a rich history in philosophy and psychology [1,2,3,4,5,6,7,8,9,10]. Traditional theories of visual perception posited that object detection (i.e., figure assignment) must occur before shape and meaning are activated. Against these views, it is now well established that both shape memory and meaning are activated before object detection, and that familiar shape is an object prior [11] (for review see [12]). Recent research indicates that presenting valid object labels (i.e., words) before objects can aid object detection [13,14,15,16,17]. This research has been taken as evidence that semantic expectations influence object detection because words and objects are connected via semantics. The mechanisms of these semantic effects are unknown, however. One possibility is that words generate predictions regarding the low-level features of the objects they denote [14,18]; these predictions would require more revision following an invalid than a valid label, thereby interfering with object detection. Alternatively, labels may activate higher-level basic-level representations of objects via connected semantic networks [19,20,21,22,23]. On this view, semantics may exert an influence at a higher level where object configurations are represented.

To understand the mechanism, it is important to investigate whether participants’ responses index object detection or feature detection in experiments suggesting semantic influences. Unfortunately, clear distinctions often cannot be made. Lupyan and Ward (2013) demonstrated that their results indexed feature detection at least and ruled out a response bias explanation [15]. While making this valuable contribution, they did not attempt to distinguish between feature detection and object detection. Yet, because feature detection is not an acceptable index of object detection, it is important to use manipulations that can discriminate between responses to features vs. objects. A standard method, not employed often in this literature (but see [24]), is to use objects that have a typical upright orientation and to present them both upright and upside down (i.e., inverted). The 180° rotation from upright to inverted keeps features intact but renders the configuration of features and parts unfamiliar [11,25,26].

To understand the mechanisms of label effects it is also important to distinguish whether valid labels increase detection accuracy, invalid labels reduce accuracy, or whether both effects occur. Comparisons of object detection accuracy following valid versus invalid labels are not sufficient for this goal. Control conditions are necessary, yet these were often absent in previous research or differences went unnoted [15,16,17].

A third important consideration is whether previous experiments have assessed object detection per se. Operational definitions and stimulus repetition are important here. Many of the relevant experiments operationalized object detection as the time at which an object broke through continuous flash suppression (b-CFS, [27]; e.g., [13,15,16,17]). In our view, emergence from suppression is not a good operational definition of object detection per se for many reasons: (1) interocular suppression must be overcome in order for CFS breakthrough to occur [28,29,30], whereas simple object detection does not entail overcoming suppression; (2) it takes a long time to break through CFS (e.g., up to 3400 ms in [15] and 3600 ms in [16]), attesting to the complex processes involved and suggesting there may have been many breakthrough opportunities before the successful breakthrough; and (3) responses in CFS experiments may index the emergence of features or parts rather than configured objects [15].

In addition to these concerns, b-CFS experiments and experiments using other methods presented the stimuli and the label-object pairs multiple times [15,16,17]. Expectations established for objects encountered repeatedly may differ from those established for objects presented only once in an experiment. With repetition, participants may rely on properties that allow them to distinguish the target object from distractors; these properties may be necessary for responses within the experimental context, but not for object detection per se.

## 2. The Present Experiments

In the present experiments, we investigated whether semantic expectations influence object detection while addressing the questions raised in the previous section. We added a control condition and presented stimuli once only. To assess object detection per se, we measured figure assignment responses. Figure assignment is one possible outcome of visual processes assessing how to interpret the two abutting regions in the visual input that share a border. When figure assignment occurs, a figure (i.e., an object) is perceived on one side of the border and a locally shapeless background is perceived on the other side. (Other outcomes are possible. For instance, the border could be perceived as a joint between two slanted surfaces, the boundary between two colored areas on a two-dimensional surface, or a shadow border). Figure assignment is archetypal object detection because before borders are assigned, patterns are present, but objects are not [12,31,32,33]. 

**Figure 1:**
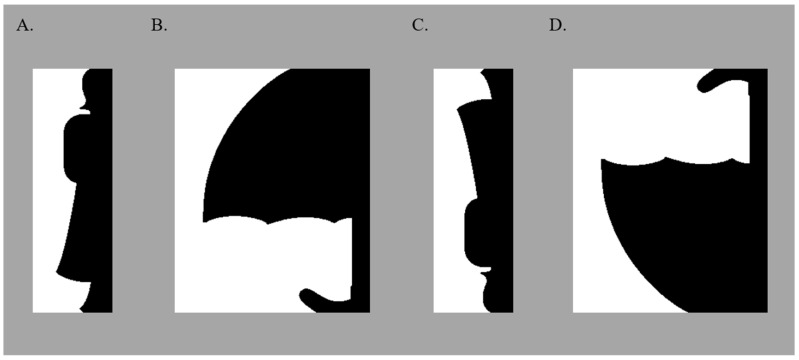
Sample Bipartite Displays. In all stimuli, a portion of a well-known object was sketched on one “critical” side of the central border; this critical region was equally often on the left/right, in black/white, and upright/inverted. In these samples, the portions of the well-known objects are sketched on the right side of the central border in black (upright portions of a woman and an umbrella are shown in (**A**,**B**), respectively; inverted versions are shown in (**C**,**D**), respectively). Displays were presented on a medium gray background.

We used bipartite stimuli like those in Figure 1, in which a central border divided a vertically elongated rectangular field into two equal-area regions. The border sketched a portion of a real-world object on one side: henceforth, the “critical side” of the border. When the border is assigned to the critical side, the real-world object sketched on that side of the border is detected. The real-world object in our displays was a member of a basic-level category with which participants were likely to be familiar (e.g., dog, tree, lamp, umbrella, etc.). The depicted instance of the basic-level category was novel, however; it was a schematic shape created to be identifiable (see Methods) yet crafted to maintain equal-area regions on opposite sides of the border.

Peterson and Gibson ([34,35,36]) previously showed that figures/objects were more likely to be detected on the critical side of the border when bipartite displays were presented such that the object was sketched in its typical upright orientation rather than an inverted orientation (cf. [11,37,38,39,40]). With the 180° orientation change from upright to inverted, the object configuration changed from typical to atypical whereas the features did not change (e.g., convexities into or out of the critical regions and the degree to which borders are curved versus straight remain constant). The orientation dependency revealed that activation of a representation of a familiar configured object was necessary for the results; features alone were not sufficient. Because no other known figure/object priors differed on the two sides of the central border, these results revealed that familiar configuration is a prior for figure assignment (We use the term “familiar configuration” to refer to configurations that are familiar at a basic level, not to configurations repeated within an experiment).

To explain the orientation dependency of the familiar configuration effects, Peterson and colleagues appealed to a proposal by Ashbridge et al., (2000)—that evidence accumulates faster in a neural population representing an object when the object appears in its typical upright orientation rather than in an inverted orientation [41] (cf. [12,42,43]). Ashbridge et al. showed that the cumulative response in such a neural population at any point in time would be larger for objects viewed in their typical upright orientation. With respect to the question of interest here—whether semantic activation can influence object detection—we point out that basic-level object representations activated by words within a semantic network (e.g., [19,20,21,23]) could take the form of neural populations like those proposed by Ashbridge et al [41]. In that case, labels would be expected to exert a larger effect on object detection in upright than inverted displays.

In the two studies reported here, bipartite stimuli sketching a familiar configuration on one side of the central border were preceded by a valid or an invalid label and masked immediately afterwards (labels-present experiments). Half the displays were preceded by valid labels; the other half were preceded by invalid labels. Valid labels denoted the objects at a basic level. Invalid labels denoted objects that were semantically unrelated to the object sketched in the display. In control experiments, no labels preceded the bipartite displays; presentation conditions were otherwise the same (labels-absent experiments). Participants reported whether they detected an object on the left (L) or right (R) side of the central border. Previous research has shown that object identification is neither necessary nor sufficient for detecting an object on the familiar configuration side of the border in these displays [39,40]. Therefore, the detection responses indexed via figure assignment are not confounded by identification. Results obtained in the labels-present experiments will be compared to those obtained in the control labels-absent experiments to explicate whether object detection accuracy is improved after valid labels, is reduced after invalid labels, or whether both effects occur.

Exposure duration was manipulated between experiments: bipartite displays were presented for 90 ms or 100 ms. Albeit only slightly longer than 90 ms, the 100-ms exposures allow more time for semantics to be activated by the object in the test display (see [19,44,45,46,47]). The 100 ms exposure also allows more time before the mask interferes with reentrant processing, which is necessary to ground the activation initiated by the target object on the left or right side of the display. Longer exposure durations were not used because object detection would be close to ceiling.

In each experiment, half the stimuli depicted familiar configurations in their upright orientation; the other half depicted inverted versions of familiar configurations. An individual participant viewed each word and each display once only. We reasoned that if semantic expectations operate via low-level feature predictions in these conditions, then label effects should be orientation independent because the amount of time needed to revise incorrect feature predictions should not vary with orientation. On the other hand, if semantic expectations operate by pre-activating higher-level representations of configured objects in these conditions, then effects should be larger for upright displays than inverted displays because at any point in time, activation in neural populations representing the objects will be larger for upright than inverted objects.

Three studies are presented: a control study and two labels-present studies. Each study included four experiments (an original and a replication experiment at each exposure duration). In labels-present experiments in Study 1, the invalid labels denoted objects in a different superordinate-level category in that labels denoting artificial objects preceded displays in which critical regions depicted natural objects, and vice versa. In Study 2, invalid labels denoted objects in the same superordinate-level category in that labels denoting artificial objects preceded displays in which critical regions depicted artificial objects and labels denoting natural objects preceded displays in which critical regions depicted natural objects. (In both studies, the invalid labels were semantically unrelated to the target objects. See Appendix A for lists of stimuli and labels).

Differences between the effects of invalid labels in Studies 1 and 2 will be informative regarding the extent to which semantic expectations operate via low-level feature predictions or by activating higher-level representations of configured objects. The features of objects in different superordinate-level (DSC) categories differ at a coarse level (e.g., predominance of straight vs. curved borders), whereas those in the same superordinate-level (SSC) category are similar at this coarse level (e.g., [48,49,50]). Therefore, if label effects are instantiated as low-level feature predictions, fewer revisions to predictions would be necessary in Study 2 (SSC invalid labels) than in Study 1 (DSC invalid labels), and response times should be lower in Study 2 than in Study 1. On the other hand, a conflict may emerge at a higher level where different neural populations are activated by the invalid label and the test object in the display. Because same-category objects are represented closer in semantic and neural space (cf. [51,52,53]), the time required to resolve the conflict (and hence, response times) would be longer in Study 2 than Study 1.

## 3. Materials and Methods

### 3.1. Participants

Participants were 458 undergraduate students (18–36 years old; M = 19.21, SD = 1.85) at the University of Arizona (UA) who took part to partially fulfill course requirements or in exchange for payment. Participants took part in one experiment only. Before the experiment, they provided informed consent and demonstrated normal or corrected-to-normal visual acuity. Data were analyzed from only those participants who met four a priori criteria that are standard in our laboratory: They (1) had least 85% usable trials; trials were deemed unusable if no response was made before timeout or if the response time (RT) was less than 200 ms; (2) reported sufficient sleep the night before the experiment; (3) primarily reported their first percept (i.e., they estimated reporting their second percept on ≤20% of trials; and (4) their mean in any condition was within two standard deviations of the condition mean (those whose means were more than two standard deviations from the condition mean were “outliers”).

A total of 130 participants (28.38%) were dropped due to failing to meet the four a priori criteria. For details, see Supplementary Materials.

Using best practices, an original and a replication experiment were conducted with each exposure duration in each Study (4 experiments per Study). Between-experiment ANOVAs showed no difference between the results of the original and replication experiments. Hence, we present the combined results here. The results of the original and replication experiments can be found in Supplementary Materials.

#### 3.1.1. Control Experiments

Accuracy data were analyzed for a total of 62 participants (RTs for 59) in the combined 90 ms experiments and for 59 participants (RTs for 54) in the combined 100 ms experiments. The number of participants varies between accuracy and RTs because outlier analyses were conducted separately. The data from individuals who were flagged as outliers in accuracy were automatically removed from the RT analysis. The number of participants varies between experiments because the outlier analyses were conducted after the goal *N* was reached and the SARS-CoV-2 pandemic prevented testing additional participants.

#### 3.1.2. Study 1: Invalid DSC Labels

Accuracy data were analyzed for a total of 54 participants (RTs for 51) in the combined 90 ms experiments and 58 participants (RTs for 54) in the combined 100 ms experiments.

#### 3.1.3. Study 2: Invalid SSC Labels

Accuracy data were analyzed for a total of 56 participants (RTs for 51) in the combined 90 ms experiments and 57 participants (RTs for 52) in the combined 100 ms experiments.

### 3.2. Apparatus and Stimuli

A Dell Optiplex 9020 computer with an Intel^®^Core™ i7-4790 CPU running at 3.60 GHz and an AOC G2460PG 24 Class Nvidia G-Sync LCD gaming monitor running at 100 Hz were used in all experiments. Participants viewed the monitor from a distance of 100 cm; head position and viewing distance were maintained by a chinrest. They used a foot pedal to advance through the instructions and to initiate each trial. Participants’ responses were recorded using a custom-made button box. Stimuli were presented using the software DMDX [54].

### 3.3. Test Displays

The test stimuli in all experiments were 72 bipartite displays (36 upright and 36 inverted; see Figure 1). In upright displays, a familiar configuration was sketched on the critical side of the border in its typical upright orientation. Half of the familiar configurations were portions of natural objects (e.g., a woman); the other half were portions of artificial objects (e.g., an umbrella) (see Appendix A for a list of the objects portrayed on the critical side of the border.) The other side, the complementary side, depicted a novel shape. Inverted displays were created by rotating upright displays by 180° and mirroring them across the vertical axis. All bipartite displays were viewed by individual participants once only. The displays, their image statistics, and normative data regarding the familiar objects they portrayed are available online at https://osf.io/j9kz2/ (accessed on 6 June 2021) (cf. [55]).

### 3.4. Labels

The labels (*N* = 108) were chosen such that three labels—Valid, Invalid (DSC), and Invalid (SSC)—were paired with each of the 36 well-known objects sketched in the bipartite displays. Valid labels denoted the object sketched in the display at a basic level. Invalid DSC labels denoted a semantically unrelated object in a different superordinate-level category (natural vs. artificial categories) and Invalid SSC labels denoted an unrelated object in the same superordinate-level category. Invalid DSC labels were used in Study 1, whereas Invalid SSC labels were used in Study 2. The same Valid labels were used in the two experiments. Individual participants viewed a label once only.

### 3.5. Design and Procedure

Participants first read and signed a consent form approved by the Human Subjects Protection Program at the University of Arizona. Next, their visual acuity was tested using a Snellen eye chart.

Participants were instructed on the nature of figure–ground perception and shown a few examples of closed bounded figures on colored backgrounds. They were then introduced to black and white bipartite displays on medium gray backgrounds and were told that their task was to report whether they perceived a figure on the left or the right side of the central border. Participants were informed that there were no correct or incorrect answers for the figure judgment task; they were instructed to report their first impression. Participants made their figure reports with their dominant hand on a response box with two horizontally aligned buttons; they pressed the right button to indicate they perceived the figure on the right side of the central border and the left button to indicate they perceived the figure on the left side of the central border. Assignment of the left and right buttons did not change across participants as “left” and “right” have intrinsic meaning with respect to the displays and all factors were balanced across the left/right sides of the displays. Participants were not encouraged to respond quickly; however, they were told their responses would be recorded only if they were made before the fixation cross for the next trial appeared (4000 ms later). RTs to make the figure reports were recorded from the onset of the bipartite test display; hence, they include the exposure durations of the test display (90 or 100 ms) and the 200 ms mask. Participants were encouraged to ask questions during both the instructions and subsequent practice trials.

### 3.6. Trial Structure

**Figure 2:**
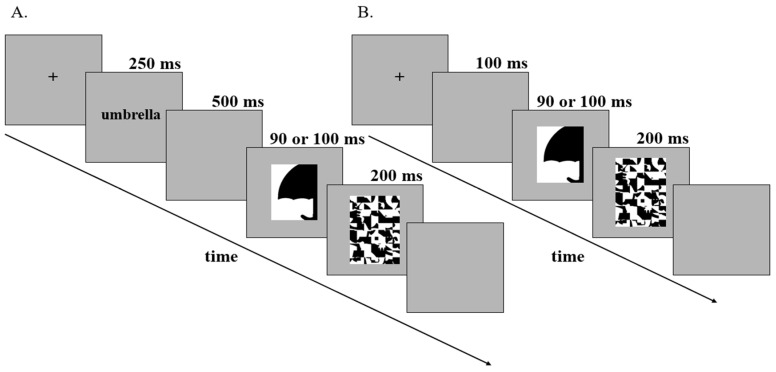
Trial Structures for labels-present and labels-absent experiments. (**A**) Trial structure for labels-present experiments (Studies 1 and 2). Following fixation, a label was displayed for 250 ms. The label was either valid or invalid. Valid labels denoted the object sketched in the displays at a basic level (e.g., “umbrella,” as depicted above); Invalid DSC labels (Study 1) denoted an unrelated object in a different superordinate-level category (e.g., “squirrel”); and Invalid SSC labels (Study 2) denoted an unrelated object in the same superordinate-level category (e.g., “envelope”). After a 500 ms blank screen, the test display was shown for either 90 ms or 100 ms (these durations were tested in separate experiments) and was followed by a 200 ms mask. The test display shown above depicts a portion of an upright umbrella sketched on the right side of the central border in black. During the experiments, the portions of common objects sketched on the critical sides of the borders were shown equally often on the left/right, in black/white, and upright/inverted. Task: Report the side on which they perceive a figure. The last, blank, screen was shown until response or 4 sec (timeout). (**B**) Trial structure for labels-absent experiments (control). Following fixation, a blank screen was displayed for 100 ms. As in labels-present experiments, the test display was shown for either 90 ms or 100 ms (durations tested separately) and was followed by a 200 ms mask. During the experiments, the portions of common objects sketched on the critical sides of the borders were shown equally often on the left/right, in black/white, and upright/inverted. Task: Report the side on which they perceive a figure. The last, blank, screen was shown until response or 3 s (timeout).

The trial structures for the experiments are shown in Figure 2. Each trial began with a central fixation cross. Participants were instructed to fix their eyes on the cross and to press the foot pedal when they were ready to initiate a trial. After the foot pedal press, in labels-present experiments in Figure 2A, a label appeared in the center of the screen (250 ms), followed by a blank screen (500 ms), then by the bipartite display (90 ms or 100 ms; these durations were tested in separate experiments), and finally by a mask (200 ms). The label was either valid or invalid (DSC) in Study 1 and either valid or invalid (SSC) in Study 2. The trial structure in control experiments in Figure 2B was similar, except that no labels were presented. Participants had 4000 ms from the onset of the test display to make their response; RTs ≥ 4000 ms were considered timeouts.

Within each study, trials were presented over two hidden blocks; 36 of the bipartite displays were shown once per block. Half of the test displays in each block were “upright” in that the object depicted on the critical side of the border was shown in its typical upright orientation. The other half were inverted, in that the object depicted on the critical side of the border was shown rotated 180° from its typical upright orientation. Upright and inverted versions of a given stimulus were presented in different blocks. Black/white contrast and left/right location of the critical side of the border were balanced and nested under orientation. Half of the stimuli in each orientation in each block were preceded by a valid label; the other half were preceded by an invalid label. The contrast and side of the critical region relative to the central border and the label-type pairing (valid vs. invalid) were changed in the second block. A total of 16 programs was used to present all combinations of orientation, label-type, contrast, and side; individual participants viewed one program only.

Before the experimental trials, participants completed 8 practice trials. None of the labels, test displays, or masks used in the practice were used in experimental trials. Participants were told that a word would appear on every trial but were not informed about the relationship between the words (labels) and the displays.

## 4. Data Analysis Methods

In each experiment we analyzed (1) accuracy indexed by the percentage of trials on which participants reported perceiving the figure on the critical side of the border where the familiar configuration was sketched; and (2) response times (RTs) for trials on which participants accurately detected the object on the critical side of the border. The bipartite displays were designed so that if participants have a bias for one side (L or R) or one contrast (black or white), objects will not be detected on the critical side of the border more often than chance; hence, the accuracy floor is 50%.

To examine performance in the labels-present experiments, we used two 2 (Orientation: upright vs. inverted) × 2 (Label Type: valid vs. invalid) × 2 (Duration: 90 ms vs. 100 ms) ANOVAs, one for accuracy and one for RTs. Exposure duration effects were obtained in only a few instances; these few instances are marked. To determine whether valid labels improved detection accuracy or invalid labels reduced detection accuracy or whether both effects occurred, we compared the accuracy and RT results obtained in the labels-present experiments to those obtained in the labels-absent control experiments in 2 (Labels: present vs. absent) × 2 (Orientation: upright vs. inverted) × 2 (Duration: 90 ms vs. 100 ms) ANOVAs. Comparisons to the control were conducted separately for valid labels and invalid labels.

## 5. Results

### 5.1. Control Experiments: 90- and 100-ms Display Durations

#### 5.1.1. Detection Accuracy

**Figure 3:**
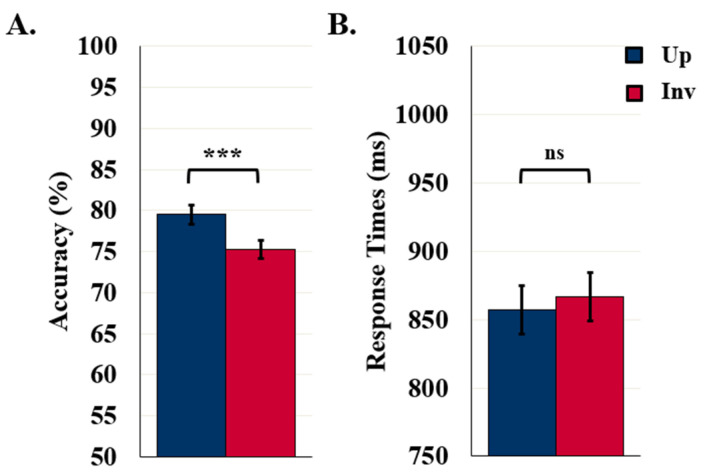
Results for control/labels-absent experiments. (**A**) Object Detection Accuracy (*N* = 121) and (**B**) Detection RTs (*N* = 113). Error bars represent standard errors. *** indicates *p* < 0.001 and ns indicates non-significance.

Detection accuracy was higher for upright (79.5%) than inverted (75.3%) displays, *F* (1119) = 28.02, *p* < 0.001, *η*^2^ = 0.19, as seen in Figure 3A. These results confirm that, for these stimuli, familiar configuration effects are mediated by representations of configured objects. Detection accuracy was also higher when the displays were exposed for 100 ms (79.8%) rather than 90 ms (75%), *F* (1119) = 5.32, *p* = 0.023, *η*^2^ = 0.04 (not shown). Orientation and duration did not interact, *F* (1119) = 1.42, *p* = 0.235.

#### 5.1.2. Response Times

The mean RT in the control experiments was 861.9 ms. RTs did not differ with exposure duration or orientation, *F* (1111) = 0.53 and 1.81, respectively, *p*s > 0.181. The absence of an orientation effect in RTs (as seen in Figure 3B) was not surprising as orientation effects have not previously been observed in RTs.

### 5.2. Study 1: Invalid DSC Labels

#### 5.2.1. Detection Accuracy

**Figure 4:**
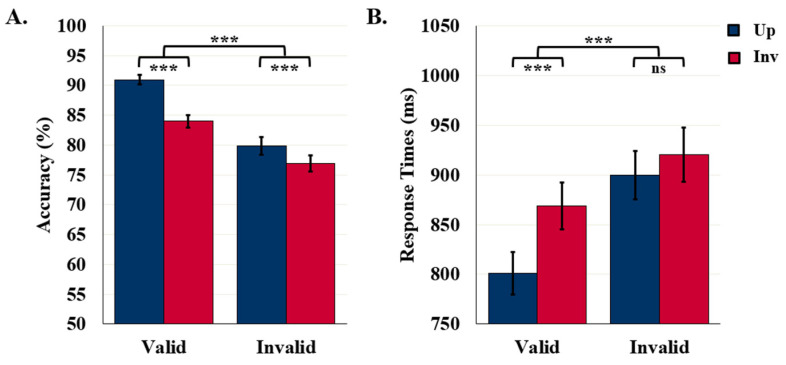
Results for Study 1: Invalid DSC Labels. (**A**) Object Detection Accuracy (*N* = 112) and (**B**) Detection RTs (*N* = 105). Error bars represent standard errors. *** indicates *p* < 0.001 and ns indicates non-significance.

Object detection accuracy was higher for upright (85.4%) than inverted (80.5%) displays, *F* (1110) = 41.08, *p* < 0.001, *η*^2^ = 0.27, affirming that these results index object detection rather than feature detection. Detection accuracy was higher following valid (87.5%) than invalid (DSC) labels (78.4%), *F* (1110) = 63.82, *p* < 0.001, *η*^2^ = 0.37. Although the upright advantage was present in both label conditions, it was larger in the valid-label condition than in the invalid-label condition, *F* (1110) = 9.30, *p* = 0.003, *η*^2^ = 0.08, as can be seen in Figure 4A. Although the orientation-dependent accuracy difference was present in both exposure duration conditions, it was larger when displays were presented for 90 ms (85.7% vs. 79.2%) rather than 100 ms (85.1% vs. 81.7%) before the mask, *F* (1110) = 3.95, *p* = 0.049, *η*^2^ = 0.04 (not shown in Figure 4). No other main effects or interactions were statistically significant.

#### 5.2.2. Response Times

Accurate object detection was faster for displays following valid (834.9 ms) rather than invalid DSC (909.8 ms) labels, *F* (1103) = 53.23, *p* < 0.001, *η*^2^ = 0.34. RTs were shorter for upright (850.2 ms) than inverted (894.5 ms) displays, *F* (1103) = 26.45, *p* < 0.001, *η*^2^ = 0.20. As seen in Figure 4B, the orientation difference was present following valid labels (upright: 800.8 ms vs. inverted: 869.0 ms), *p* < 0.001, but not following invalid DSC labels (upright: 899.5 ms vs. inverted: 920.1 ms), as revealed by an interaction between orientation and label type, *F* (1103) = 7.87, *p* = 0.006, *η*^2^ = 0.07.

#### 5.2.3. Comparison of Study 1 Results to Control Results

Next, to elucidate whether valid labels increased and/or invalid labels decreased object detection speed and accuracy, we compared the accuracy and RTs obtained in the labels-present experiments to those obtained in the control experiments.

##### Accuracy 

**Figure 5:**
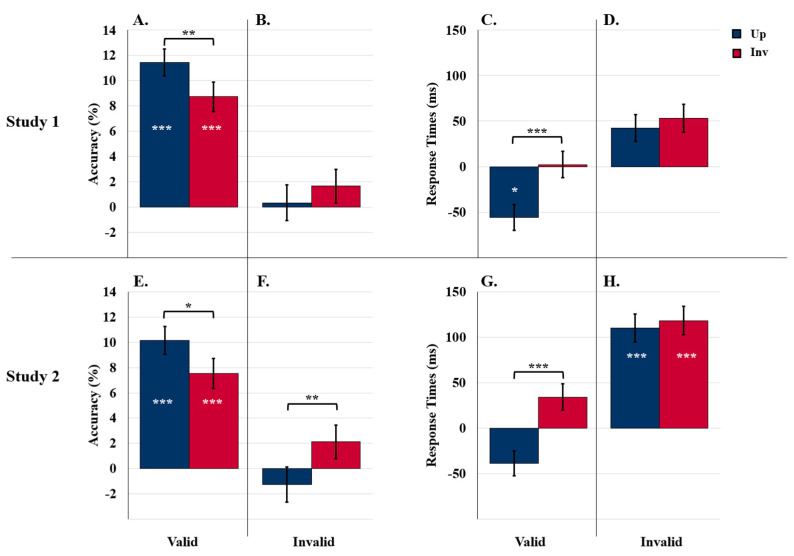
Results for comparisons to control. (**A**–**D**) Comparisons to Study 1 and (**E**–**H**) comparisons to Study 2. Object detection accuracy difference scores following valid (**A**,**E**) and invalid (**B**,**F**) labels. Detection RT differences scores following valid (**C**,**G**) and invalid (**D**,**H**) labels. White asterisks indicate main effects of condition (experimental vs. control). Brackets and black asterisks indicate two-way interactions between conditions (experimental vs. control) and orientation. Error bars represent pooled standard errors. *** indicates *p* < 0.001, ** indicates *p* < 0.03, and * indicates *p* < 0.05.

The valid-labels ANOVA revealed a main effect of label presence: accuracy was higher than control when labels were present (87.5% vs. 77.4%), *F* (1229) = 58.00, *p* < 0.001, *η*^2^ = 0.20. As seen in Figure 5A, the increase due to the presence of the label was larger for upright than inverted displays (11.6% vs. 8.8%), as revealed by an interaction between label presence and orientation, *F* (1229) = 4.89, *p* = 0.028, *η*^2^ = 0.02. This orientation-dependent effect was present in both exposure duration conditions.

The invalid-labels ANOVA revealed an interaction between orientation, exposure duration, and label presence, *F* (1229) = 7.25, *p* = 0.008, *η*^2^ = 0.03. This three-way interaction (not shown in Figure 3B) was obtained because the upright advantage in detection accuracy was present in the 90-ms experiments but not in the 100-ms experiments following an invalid label, *t* (57) = 0.17, *p* = 0.864. Thus, the comparison to the control revealed that invalid labels can reduce object detection accuracy, at least when target objects are upright and displays are presented for 100 ms.

##### Response Times 

As can be seen in Figure 5C, the valid-labels ANOVA revealed an interaction between orientation and label presence, *F* (1214) = 20.22, *p* < 0.001, *η*^2^ = 0.09. Response times for upright displays were faster than control when valid labels were present (800.8 ms vs. 857.1 ms; difference = −56.3 ms), *p* = 0.044, but response times for inverted displays were unaffected (869.0 ms vs. 866.7 ms; difference = 2.3 ms), *p* = 0.924. There was no effect of exposure duration, *p* = 0.542. Thus, upright, but not inverted, objects were detected faster following valid labels than no labels.

The invalid-labels ANOVA revealed only a main effect of orientation unmodulated by label presence vs. absence or exposure duration: response times were faster for upright (878.3 ms) than inverted (893.4 ms) displays, *F* (1214) = 4.27, *p* = 0.040, *η*^2^ = 0.02. Differences from the control for upright and inverted displays (42.3 ms vs. 53.3 ms) can be seen in Figure 5D.

#### 5.2.4. Study 1 Summary

In the labels-present experiments in Study 1, we found both label type and orientation effects. Orientation effects were evident in comparisons to the control as well, indicating that, at least in the current experiments, labels do not generate predictions regarding the features of the objects they denote. The stimuli were designed such that object features do not change with the 180° change in orientation; orientation-independence is expected if the results are due to predictions regarding features. Instead, the comparison to the control indicated that label effects were orientation dependent: RTs for upright displays were faster than control when valid labels were present, but RTs for inverted displays were unaffected by labels. Similarly, the label effects evident in accuracy were larger for upright than inverted objects. This was true both for the positive effects of valid labels and for the negative effect of invalid labels observed in the 100 ms experiments.

Thus, the Study 1 results are better fit by the hypothesis that labels pre-activate the neural population representing the object they denote [56], one in which evidence accumulates faster for upright than inverted objects (cf. [41]). Invalid labels activate a different neural population. The pre-activation of a different neural population by an invalid label eliminated the upright advantage in the 100 ms exposure condition only. This condition is one in which the semantics of the upright target object was expected to be most highly activated, because the longer exposure duration allows more time for the semantics to be activated. Semantic activation initiated by the upright target object in the 100 ms condition may be high enough to conflict with the semantics of the different-category object pre-activated by the invalid label. This conflict may disrupt object detection. We investigate the hypothesis of semantic conflict between the neural populations activated by the label and the target object in the display further in Study 2.

### 5.3. Study 2: Invalid SSC Labels

In Study 1, invalid labels denoted semantically unrelated objects in a different superordinate-level category (DSC) from the target object. In Study 2, invalid labels denoted a semantically unrelated object in the same superordinate-level category (SSC) as the target object. This change allowed us to further investigate the hypothesis that feature expectations produce the label effects. The features of objects in the SSC are similar to those of the target object. Therefore, if labels initiate predictions regarding low-level features of objects that must be revised when not confirmed by the input, the effects of invalid labels should be reduced in Study 2. This is because smaller prediction revisions would be required following SSC rather than DSC invalid labels. In contrast, because the neural populations representing SSC objects lie closer to each other in semantic and neural space (e.g., [51,57,58,59,60,61,62,63,64,65]), conflict between them should be larger.

#### 5.3.1. Detection Accuracy

**Figure 6:**
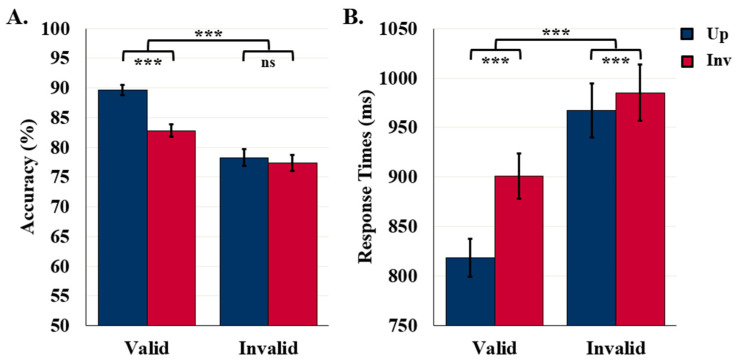
Results for Study 2: Invalid SSC Labels. (**A**) Object Detection Accuracy (*N* = 113) and (**B**) detection RTs (*N* = 103). Error bars represent standard errors. *** indicates *p* < 0.001 and ns indicates non-significance.

Detection accuracy was higher following valid (86.2%) rather than invalid SSC labels (77.8%), *F* (1111) = 58.97, *p* < 0.001, *η*^2^ = 0.35. A main effect of orientation was observed, but an interaction between orientation and label type, in Figure 6A, showed that the upright advantage was present in the valid-label condition (up-inv difference = 6.9%), but absent in the invalid (SSC)-label condition (up-inv difference = 0.9%, *F* (1111) = 13.99, *p* < 0.001, *η*^2^ = 0.11. Object detection accuracy was higher when displays were exposed for a longer duration before the mask: 100 ms (84.3%) vs. 90 ms (79.8%), *F* (1111) = 6.11, *p* = 0.015, *η*^2^ = 0.05), but there were no interactions involving duration, *p*s > 0.356.

#### 5.3.2. Response Times

Response times were faster for upright (892.8 ms) than inverted (943.1 ms) displays, *F* (1101) = 23.47, *p* < 0.001, *η*^2^ = 0.19; and for displays preceded by valid (859.7 ms) rather than invalid SSC (976.2 ms) labels, *F* (1101) = 81.81, *p* < 0.001, *η*^2^ = 0.45. An interaction between label type and orientation indicated that the upright advantage in RTs was smaller for invalid (SSC) labels than for valid labels (up-inv difference = 17.8 ms vs. 82.6 ms), *F* (1101) = 10.45, *p* = 0.002, *η*^2^ = 0.09, although a significant upright advantage was observed in both conditions, *p*s < 0.001, as seen in Figure 6B. No effect of exposure duration was observed, *p* = 0.970.

#### 5.3.3. Comparison of Study 2 Results to Control Results

Next, as in Study 1, we compared the accuracy and RTs obtained in the labels-present experiments to those obtained in the control experiments in order to elucidate whether valid labels increased and/or invalid labels decreased object detection speed and accuracy.

##### Accuracy

The valid-labels ANOVA revealed a main effect of label presence: accuracy was higher than control when valid labels were present (86.2% vs. 77.4%), *F* (1230) = 42.68, *p* < 0.001, *η*^2^ = 0.16. As seen in Figure 5E, the increase due to the presence of the label was larger for upright than inverted displays (10.1% vs. 7.5%), as revealed by an interaction between orientation and label presence, *F* (1230) = 4.31, *p* = 0.039, *η*^2^ = 0.02. Effects of valid labels did not vary with exposure duration. Thus, as in Study 1, valid labels had a positive influence on detection accuracy that was larger for upright displays in both exposure durations.

The invalid-labels ANOVA revealed an interaction between label presence and orientation, *F* (1230) = 5.71, *p* = 0.018, *η*^2^ = 0.02: As seen in Figure 5F, the typical upright advantage observed in the control experiments was not observed following invalid SSC labels (advantage = 0.9%) (this effect is not surprisingly given the absence of an orientation effect following invalid labels in the labels-present ANOVA). Exposure duration did not interact with this orientation effect, *p* = 0.99. Thus, when displays were preceded by invalid SSC labels, the upright advantage in accuracy was eliminated in both exposure duration conditions.

##### Response Times

The valid-labels ANOVA confirmed that, in contrast to the control condition that showed no RT advantage for upright displays, an upright advantage of 82.6 ms emerged in the valid label condition, *F* (1212) = 29.84, *p* < 0.001, *η*^2^ = 0.12. Thus, as in Study 1, detection RTs are speeded for upright relative to inverted target objects following a valid label.

The invalid-labels ANOVA revealed a main effect of label presence: Accurate detection was substantially and significantly slower than control following invalid SSC labels, *F* (1212) = 13.38, *p* < 0.001, *η*^2^ = 0.06. No other main effects or interactions were significant, *p*s > 0.122. As seen in Figure 5H, for target objects in both orientations, accurate detection RTs were slower than control when displays followed invalid SSC labels (upright difference: 110.2 ms; inverted difference: 118.4 ms).

Once again, comparisons to control show that the effects of both valid and invalid labels on detection accuracy were orientation dependent, contrary to what would be expected if feature predictions were the mechanism of the label effects. In Study 2, the upright advantage in accuracy was enhanced by valid labels and eliminated by invalid SSC labels; this effect was observed in both exposure conditions. Because the semantic distance is smaller from target objects to SSC objects than to DSC objects (e.g., [49,66]), greater conflict was expected to occur between the neural populations representing target objects in the test displays and objects denoted by invalid labels in Study 2 than in Study 1. In Study 2, conflict in the invalid labels condition affected detection accuracy for upright objects earlier in time (e.g., 90 ms displays). A temporally earlier effect is consistent with greater conflict.

The comparison of control RTs to RTs obtained when labels were present buttresses the conflict interpretation. In Study 2, when invalid SSC labels preceded the displays, RTs were longer than the control by >100 ms for both upright and inverted displays, with no effect of exposure duration. Given that the accuracy results support the interpretation that, in our experiments, labels operate by activating the neural populations representing the objects they denote, we interpret the RT results in terms of activated neural populations. Because we analyzed RTs for accurately detected objects only (the percentage of inaccurate detection responses was too small to support a sensitive statistical analysis) and accuracy was high, we assume that the neural populations representing the target objects were activated strongly enough to enable successful reentrant processes. In Study 2, that activation was sufficiently strong so that semantic conflict emerged between the different neural populations representing the target object and the object denoted by the invalid SSC label. The conflict eliminated the upright advantage in accuracy in both exposure durations and increased RTs in both orientations in Study 2. The latter finding is consistent with the idea that the semantic network representing an object includes object properties in addition to object shape and object labels [23]. Therefore, it is not surprising that semantic conflict can be seen between object properties/features present in both orientations. The orientation independency of these RT results situates the conflict in the semantic system; it does not require a revision of the attribution of the orientation dependency of the valid label effects to the pre-activation of a neural population representing the denoted object. Thus, the results of Study 2 show that, even when detection responses are accurate, they are slower when there is conflict in the semantic system. It takes time to resolve competition (e.g., [67,68,69]). These results are consistent with the interpretation that detection responses are delayed until conflict in the semantic system is resolved.

## 6. Comparing Study 1 and Study 2

For completeness, we compared the results of Study 1 and Study 2 directly. Here, we report only between-study differences. The results of this analysis can be seen by comparing Figure 4 and Figure 6.

### 6.1. Detection Accuracy

No differences between the accuracy results of Study 1 and Study 2 were observed, *F* (1221) = 0.49, *p* = 0.482.

### 6.2. Response Time

The ANOVA comparing RTs in Studies 1 and 2 yielded an interaction between study and label type, *F* (1204) = 6.41, *p* = 0.012, *η*^2^ = 0.03. Accurate detection RTs following valid labels did not differ statistically between the two studies (difference = 24.8 ms), whereas accurate detection RTs were 66.4 ms longer following invalid labels in Study 2 (976.2 ms) than in Study 1 (909.8 ms). These RT results are consistent with the hypothesis that, on invalid trials in Study 2, the conflict between the neural populations representing the target object and the object denoted by the invalid label was larger when the latter was in the same rather than a different superordinate category (as in Study 1).

Recall that participants reported whether they detected an object on the left or right side of the central border. The labels convey no information about side. Therefore, long-detection RTs in Study 2 do not index a conflict between left and right responses. The conflict is semantic conflict between neural populations activated by an SSC label and by the target object. The conflict reduced accuracy only for upright target objects. The effects of conflict on accuracy, although evident for upright displays, were small because reentrant processes anchor the semantic activation initiated by the target object on the critical side of the border of the display, supporting accurate R/L responses. Accurate detection RTs were longer in Study 2 than in Study 1 in both upright and inverted conditions. Our evidence that accurate object detection is delayed by semantic conflict implies that recurrent processing determining object location with respect to the border is necessary, but not sufficient, for accurate detection of familiar configurations. When figure assignment is based on the familiar configuration prior, figure assignment responses require the resolution of semantic conflict.

## 7. Discussion

Recent research purported to show that semantics (i.e., meaning) influences object detection; semantic activation was manipulated by presenting valid or invalid labels before visual displays. Strong evidence is required to support this claim because the question of whether visual perception is influenced by meaning has a contentious history in philosophy and psychology (e.g., [1,2,3,4,5,6,7,8,9,10]). A review of the literature investigating whether labels shown before visual displays affect object detection identified a variety of methodological issues that raised the questions that were addressed here. One question was whether previous experiments assessed object detection per se as opposed to emergence from suppression or identification of degraded objects. Accordingly, we assessed object detection via figure assignment responses rather than via b-CFS or detection tasks involving degraded stimuli. The figure assignment responses are archetypal object detection because figure assignment is one outcome of processes that determine where objects lie in the visual field.

In the bipartite test displays we used, object detection was based on one figural prior-a familiar configuration was sketched on one, “critical,” side of the display. Displays were exposed briefly (90 or 100 ms) and followed immediately by a 200 ms mask. The participants’ task was to report where they detected a figure/object – on the left or right side of the display. Reports of a figure on the critical side of the test display were considered accurate responses. We assessed whether semantic activation initiated by valid or invalid labels presented before the test displays affected object detection accuracy and/or response times. Participants were told that a word would appear on every trial, but they were not informed about the relationship between the words (labels) and the displays. While it might become clear to participants over time that the displays contained a portion of a familiar object, simply looking for the object named by the label would not be a conducive strategy because (1) the labels were not predictive (50% valid, 50% invalid), (2) participants did not know which portion of an object would be depicted or how that portion would be posed, and (3) the label did not give any location information relevant to the left/right response. Furthermore, previous research has shown that object identification is neither necessary nor sufficient for detecting an object on the familiar configuration side of the border in these displays [39,40]. Therefore, the detection responses indexed via figure assignment are not confounded by identification.

A second question was whether the results of some previous experiments indexed feature detection rather than object detection. The detection of configured objects is expected to be higher when objects are shown in their typical upright orientation rather than an inverted orientation, whereas feature detection should be invariant over a change from upright to inverted. In the current experiments, as in previous experiments investigating familiar configuration effects on figure assignment, we found that detection accuracy was higher for upright than for inverted objects, demonstrating that they are configural effects rather than feature effects. We also found that labels had larger effects on object detection accuracy in upright displays than in inverted displays, demonstrating that semantic expectations initiated by labels affect object detection per se and not just feature detection. Moreover, the finding that label effects were larger for upright than inverted displays is evidence that label effects are mediated, at least in part, by higher-level neural populations representing the objects at a basic level, neural populations that are part of an interconnected semantic system.

A third question was whether repetition of the labels and objects in previous experiments may have encouraged participants to make predictions that supported target discrimination within the experiment but would not support object detection under many circumstances in the real world. While we have no doubt that low-level feature predictions can support detection following multiple repetitions of stimuli (cf. [70,71]), we were interested in how semantic expectations operate in real-world conditions where they would be more likely to operate at a basic level rather than at a subordinate level. For instance, when entering a colleague’s office for the first time, one would expect to find a desk, but not a particular desk in a particular location in their office. We were interested in uncovering the mechanisms whereby basic-level expectations affected the detection of a novel instance of a known category (like the desk). Accordingly, the labels and the test displays were presented once only in our experiments. Our results support the interpretation that under these conditions, which are like many real-world experiences, labels operate via a semantic network to activate the neural population representing the objects they denote.

A fourth question was whether valid labels improved detection, invalid labels impaired detection, or both effects occurred. Control conditions necessary to answer this question were often missing. We added control conditions and found that, following valid labels, object detection accuracy was higher and response times were decreased in an orientation-dependent manner—the effects were larger for upright objects than inverted objects. In contrast, following invalid labels, the upright advantage for accuracy was not observed when displays were exposed for 100 ms in Study 1 where DSC invalid labels were used, and in both 90 and 100 ms exposure experiments in Study 2 where SSC invalid labels were used. We submit that accuracy was impaired only enough to eliminate the upright advantage following invalid labels because the recurrent processing that anchors activation of the neural population representing the target to the right or left side of the test display is little affected by the activation of a neural population representing a different object denoted by the invalid label. Response times tell a different story: accurate detection response times were substantially longer following invalid SSC labels in Study 2 than invalid DSC labels in Study 1, revealing evidence of a conflict between the neural populations activated by invalid SSC labels and by the test displays. The conflict was expected to be greater when invalid labels denoted objects in the SSC as the target object because their representations are closer in neural and semantic space. It takes time to resolve conflict and more time for greater conflict [67,68,69]. Thus, the RT results indicate that, at least for familiar objects, detection is delayed when conflicting semantic activation is present. This is a new conclusion regarding the relationship between object detection and semantics. It shows that familiar object detection is not simply affected by semantic activation; it entails semantic activation. The detection of a familiar object, as operationalized by reports of perceiving a figure on the familiar configuration side of a border, does not occur until semantic conflict regarding which familiar object is present is resolved.

The observation of longer accurate detection RTs for both upright and inverted target objects following invalid SSC labels in Study 2 is consistent with our conclusion that semantic conflict underlies the long RTs for accurate detection. Semantic networks representing words and objects include object features and properties in addition to object shape and object labels (e.g., [20,23]). Therefore, it is not surprising that semantic conflict is evident in both orientations. The orientation independency of these RT results situates the conflict in the semantic system.

Boutonnet and Lupyan (2015) had previously concluded that words facilitated processing at low levels in the visual processing hierarchy where features are analyzed [72]. They reached this conclusion because they found effects of word primes on object recognition responses in the amplitude of the P1 component of the ERP, which is evident approximately 100 ms post-stimulus onset. Although their evidence that primes can exert an influence on perception so early in time is compelling, it is not wise to map an ERP component evident early in time onto activity in low levels of the visual processing hierarchy. Others have shown that high-level processing is indexed by the P100. For instance, Trujillo et al., ([73,74]) found higher amplitude P1 components when familiar configurations were suggested, but not consciously perceived, on the outside of the borders of their symmetric, enclosed, novel stimuli (the familiar object was not perceived because more figural priors favored the inside as the figure/object in their stimuli.) Sanguinetti et al. (2016) showed that the higher amplitude P1 responses indexed greater inhibitory competition for figure assignment when portions of familiar configurations were suggested on the outside of the border [75]. Thus, the P1 component of the ERP can index activity at levels higher than feature levels.

Applying similar reasoning, Boutonnet and Lupyan (2015) found that a later component, the N300/400, was reduced for objects that followed congruent primes and concluded that this difference indexed post-perceptual semantic activity [72]. However, the conclusion regarding post-perceptual processing does not necessarily follow. Sanguinetti et al. (2014) found reduced N300/400 responses when their stimuli followed a word denoting the object that was suggested but not perceived on the outside of their borders. Thus, Sanguinetti et al. (2014) demonstrated repetition suppression for the semantics of objects that were considered during the perceptual organization of the display but were rejected in favor of a novel object on the other side of the border [74]. These processes do not follow perception; instead, they are part of a dynamic process that chooses the best interpretation for a display. Sanguinetti et al.’s (2014) conditions were very similar to our valid and invalid label conditions, although figural priors were arranged such that the familiar configuration was not perceived as the figure. We expect that similar repetition suppression effects would be observed in the valid label conditions of the experiments reported here.

Our results are not consistent with Pinto et al.’s (2015) claim that words shown before test displays affect object detection only when they denote the object in the upcoming display on more than 50% of trials [16]; that is, only when the words have high predictive validity regarding the target object. In contrast, we found that semantic activation initiated by words influences object detection even though they were not predictive in that valid and invalid labels each appeared on 50% of trials. Therefore, contrary to Pinto et al.’s claim, semantic activation influences object detection even when labels do not predict the target, thereby implicating the semantic system in the detection of basic-level objects.

On a Bayesian model of visual processing (e.g., [76,77]), perception involves combining the current or remembered input with expectations based on past experience which take the form of previously established perceptual categories (e.g., color categories in Regier and Xu’s work). On this view, effects of expectations are evident when input is weak or ambiguous because expectations are assigned more weight under those conditions than under conditions when the input is strong and unambiguous. Previous research investigating whether semantic expectations influence object detection used degraded displays. Our displays were not degraded, nor were they ambiguous. Only one figural prior, familiar configuration, was present, and it was present on only one side of the border. The familiar configuration prior was not weak, as indexed by (1) pilot participants’ ability to identify the object depicted by the familiar configurations in our test displays (mean agreement = 89.15%); (2) by control participants’ figure reports (80% object detection accuracy in upright control displays); (3) by the conflict that emerged between the semantics of the familiar configuration and that of the object denoted by the invalid label in Study 2; and (4) by the fact that, for the most part, familiar configuration prevailed in that conflict, leaving object detection accuracy unchanged. Accordingly, although our results are consistent with a Bayesian Brain hypothesis, they cannot be accommodated by the proposal that semantic categories are weighted highly for object detection only when the input is weak or ambiguous, unlike previous experiments investigating semantic influences on object detection (e.g., [17]). Instead, our results, measured rigorously via figure assignment responses, show that the prior exposure of a valid label for an object activated the units involved in detecting that object, thereby increasing detection accuracy. Our results also show that conflict occurred in the semantic system when an invalid SSC label preceded a display depicting a familiar configuration. This conflict delayed detection responses but did not exert a large influence on detection accuracy. We conclude that the semantic system is not only involved in recognizing objects (cf. [20,21,23]); it plays a role in detecting objects.

## 8. Open Questions

One question is whether the effects reported here would be obtained if the labels were shown very briefly and were preceded and followed by masks such that observers were unaware of them. The answer depends, in part, upon how much activation of a word representation is necessary to initiate dynamic activity within the semantic system connecting word and object representations. To render words unconscious, substantially shorter exposure durations and masking are required. Semantic activation initiated by those short exposure durations may not be sufficient to initiate robust processing or broad activation of the dynamic semantic network. The answer may also depend upon whether the stimulus onset asynchrony (SOA) between the label and the display was long enough to activate the semantic network. The present studies employed a 750 ms SOA between the onset of the word and the onset of the test display. Experiments using brief masked exposures of words typically employ much shorter word-to-target SOAs. Research directed to this question is underway in our laboratory (Skocypec & Peterson, in preparation).

A second question is whether our results constitute evidence that language influences perception. Lupyan et al. (2020) make the case that the language we speak affects what we perceive [78]. Words are essential components of language, but words alone do not constitute a language; languages involve grammar and syntax as well. Although our results are not inconsistent with Lupyan et al.’s (2020) claim that language influences object perception, they stop short of demonstrating that language per se has an influence. (It could nevertheless be interesting to examine whether for bilingual speakers, words in their first and second language exert similar effects over the same time course). Ample evidence shows that words, object shape, object properties, and object meaning are represented in a distributed, interconnected, semantic network (e.g., [20,21,22,23]). Our results are best explained by activation within this dynamic interconnected network.

## Data Availability

Data supporting reported results can be found at https://osf.io/2xqz3/.

## Supplementary Materials

The following supporting information can be downloaded at: https://www.mdpi.com/article/10.3390/vision6010019/s1, Figure S1: Object Detection Accuracy and Detection RTs: Control Originals and Replications; Figure S2: Object Detection Accuracy and Detection RTs: Study 1 Originals and Replications; Figure S3: Object Detection Accuracy and Detection RTs: Study 2 Originals and Replications. References [79,80,81] are cited in Supplementary Materials.

## Appendix A

### List of Stimuli and Labels

**Stimulus/Valid Label**	**Study 1: Invalid DSC Label**	**Study 2: Invalid SSC Label**
anchor	spider	candle
axe	ant	mop
bell	fish	book
boot	frog	oven
bulb	clam	rake
butterfly	submarine	blueberry
cow	toy	log
dog	bed	hay
eagle	robot	peach
elephant	envelope	broccoli
face	door	fire
faucet	gopher	podium
flower	wallet	turkey
foot	cake	corn
guitar	turkey	needle
hand	road	fish
horse	paper	lemon
house	honey	watch
hydrant	piranha	spatula
kettle	jaguar	zipper
lamp	bush	drum
leaf	glue	swan
owl	rag	pea
palm tree	blue jean	rain drop
pig	hat	ivy
pineapple	cardboard	alligator
rhino	pedal	mango
snowman	pelican	band-aid
toilet	tongue	button
train	river	radio
tree	film	goat
trumpet	lettuce	compass
umbrella	squirrel	envelope
watering can	electric eel	baseball bat
wineglass	persimmon	thumbtack
woman	money	shark
