# Supplementary material for: Semantic Expectation Effects on Object Detection: Using Figure Assignment to Elucidate Mechanisms
Source: Vision (Basel). 2022 Mar 21;6(1):19. doi: 10.3390/vision6010019 (PMC8953613; doi:10.3390/vision6010019)
Supplement: Supplementary file 1 [file vision-06-00019-s001.zip › vision-1541621-supplementary.pdf]

Supplementary Materials

Method

Participants

Participants who took part in an experiment in exchange for payment received $10.

To meet the second a priori retention criterion, participants had to report more than four hours of sleep the night before – those who reported four or fewer hours were considered sleep deprived (sleep deprivation has been shown to reduce activation in visual processing regions (e.g., [79]) and is associated with decreased cognitive performance (e.g., [80]). Data from participants who failed to meet the first three criteria were discarded and additional participants were tested. New participants were tested in the same programs and then the outlier analysis was performed.

Of the 458 undergraduate students who participated in our experiments, we dropped 130 (28.38%) due to failing to meet the four a priori criteria. A total of 67 participants (14.63%) was dropped for failing to meet the first three criteria (8 for not having 85% usable trials, 25 for lack of sufficient sleep, 31 for reporting their second impression on ≤ 20% of trials, and 3 for both lack of sufficient sleep and reporting their second impression). A total of 38 participants (8.30%) was pulled as outliers for detection accuracy (these individuals were also pulled from the RT analysis), and an additional 25 participants (5.46%) were pulled as outliers for RTs.

Control Experiments

90-ms Exposures: Original and Replication. The participants in the *original* 90-ms exposure duration experiment were 38 undergraduate students (33 female, 5 male; 26 took part to partially fulfill course requirements). The data from one participant were discarded prior to analysis because they did not maintain fixation. One participant failed to meet the second a priori

retention criterion and four participants failed to meet the third criterion. The data from an additional participant were removed as an outlier from the accuracy analysis; that participant and an additional two were removed as outliers from the RT analysis.

The participants in the *replication* experiment were 38 undergraduate students (25 female, 13 male; 36 took part to partially fulfill course requirements). The data from one participant were discarded prior to analysis because they did not maintain fixation. Two participants failed to meet the first a priori retention criterion, two participants failed to meet the second criterion, and one participant failed to meet the third criterion. The data from an additional participant were removed as an outlier from the accuracy analysis; that participant and one more were removed as outliers from the RT analysis.

100-ms Exposures: Original and Replication. The participants in the *original* 100-ms exposure duration experiment were 40 undergraduate students (28 female, 12 male; 30 took part to partially fulfill course requirements). Five participants failed to meet the second a priori retention criterion and three participants failed to meet the third criterion. The data from an additional two participants were removed as outliers from the accuracy analysis; those two participants and an additional two were removed as outliers from the RT analysis.

The participants in the *replication* experiment were 40 undergraduate students (27 female, 13 male; 35 took part to partially fulfill course requirements). The data from one participant were discarded prior to analysis because they did not maintain fixation. Two participants failed to meet the second a priori retention criterion and five participants failed to meet the third criterion. The data from an additional three participants were removed as outliers from the accuracy analysis; those three participants and three more were removed as outliers from the RT analysis.

Study 1: Invalid DSC Labels

90-ms Exposures: Original and Replication. The participants in the *original* 90-ms exposure duration experiment were 37 undergraduate students (28 female, 9 male; 26 took part to partially fulfill course requirements). The data from one participant were discarded prior to analysis because they did not follow task instructions. One participant failed to meet the first a priori retention criterion, two participants failed to meet the second criterion, and one participant failed to meet the third criterion. The data from an additional three participants were removed as outliers from the accuracy analysis; those three participants and an additional two were removed as outliers from the RT analysis.

The participants in the *replication* experiment were 36 undergraduate students (26 female, 10 male; all 36 took part to partially fulfill course requirements). Two participants failed to meet the first a priori retention criterion, one participant failed to meet the second criterion, and one participant failed to meet the third criterion. The data from an additional seven participants were removed as outliers from the accuracy analysis; those seven participants and one additional participant were removed as outliers from the RT analysis.

100-ms Exposures: Original and Replication. The participants in the *original* 100-ms exposure duration experiment were 45 undergraduate students (28 female, 17 male; all 45 took part to partially fulfill course requirements). The data from one participant were discarded prior to analysis because they had difficulties with the English language and reported that they did not see any words. One participant failed to meet the first a priori retention criterion, five participants failed to meet the second criterion, and six participants failed to meet the third criterion. The data from an additional two participants were removed as outliers from the

accuracy analysis; those two participants and one additional participant were removed as outliers from the RT analysis.

The participants in the *replication* experiment were 35 undergraduate students (22 female, 13 male; 30 took part to partially fulfill course requirements). Two participants failed to meet the second a priori retention criterion and one participant failed to meet the third criterion. The data from an additional four participants were removed as outliers from the accuracy analysis; those four participants and an additional three participants were removed as outliers from the RT analysis.

Study 2: Invalid SSC Labels

90-ms Exposures: Original and Replication. The participants in the *original* 90-ms exposure duration experiment were 37 undergraduate students (28 female, 9 male; all 37 took part to partially fulfill course requirements). One participant failed to meet the second a priori retention criterion and four participants failed to meet the third criterion. The data from an additional four participants were removed as outliers from the accuracy analysis; those four participants and an additional three participants were removed as outliers from the RT analysis.

The participants in the *replication* experiment were 35 undergraduate students (27 female, 8 male; all 35 took part to partially fulfill course requirements). The data from one participant were discarded prior to analysis because they were familiar with our stimuli and therefore knew a portion of a real-world object was sketched on one side of the border of our displays. One participant failed to meet the second a priori retention criterion and another participant failed to meet both the second and third criteria The data from an additional four participants were removed as outliers from the accuracy analysis; those four participants and an additional two participants were removed as outliers from the RT analysis.

100-ms Exposures: Original and Replication. The participants in the *original* 100-ms exposure duration experiment were 39 undergraduate students (22 female, 17 male; all 39 took part to partially fulfill course requirements). The data from one participant were discarded prior to analysis because they did not maintain fixation. Two participants failed to meet the first a priori retention criterion and four participants failed to meet the third criterion. The data from an additional six participants were removed as outliers from the accuracy analysis; those six participants and an additional three participants were removed as outliers from the RT analysis.

The participants in the *replication* experiment were 38 undergraduate students (31 female, 7 male; 25 took part to partially fulfill course requirements). Three participants failed to meet the second a priori retention criterion, one participant failed to meet the third criterion, and two participants failed to meet both the second and third criteria. The data from an additional participant were removed as an outlier from the accuracy analysis; this participant and an additional two participants were removed as outliers from the RT analysis.

Apparatus and Stimuli

Test Displays

One region of the bipartite displays was black and the other region was white. The displays were shown on medium gray backgrounds throughout the experiment; the black and white regions contrasted equally with the background. Bipartite images subtended 5.45° H and an average of 3.49° W (range = 1.76 – 5.53°).

The normative data on the displays showed high mean agreement on the identity of the real-world object sketched on the critical side of the border of the stimuli used in this experiment (M = 89.15%; SD = 8.17) and low mean agreement for the complementary side (M = 13.98%; SD = 6.83). Together, the high agreement for the critical side and the low agreement for the

complementary side demonstrate that the familiar configuration prior is present on the critical side, but not the complementary side, of the central border.

Masks

Masks for the upright stimuli were made by randomly shuffling portions of critical regions from 12 bipartite stimuli. Starting images were created by placing critical regions, alternating in contrast from black to white, horizontally in close succession such that the number of black and white pixels was equated. Four starting images were created using the 48 stimuli tested in the norming experiment (including the 36 used in this experiment). These images were then cut into 50 x 50-pixel squares; the location of each square was randomly shuffled within each starting image and the resulting image was split at the midline to create two masks. The same process was performed on inverted starting images to create masks for the inverted stimuli. A total of 72 unique masks was used in these experiments, each was 350 X 500 pixels in height (H) and width (W), respectively. Masks subtended 5.57° H and 7.90° W.

Labels

Invalid labels were not semantically related to their paired valid labels but were matched in length and frequency; they were found using the SUBTL Word Frequency Database [81]. The word labels were displayed in lowercase Times New Roman font subtending a range of 0.29° to 0.80° H and a range of 0.80° to 3.55° W.

Design and Procedure

After the experimental trials, but before debriefing, participants were asked a series of post-experiment questions. One of the questions probed whether they reported their first percept or changed their mind and reported their second percept. Their responses to this question were

used to eliminate data from any participant who reported changing to their second percept on ≥ 20% of the trials.

Results

The graphs depicting the results of the original and replication experiments with each exposure duration in each experiment are shown in Figures S1, S2, and S3 for Control, Study 1, and Study 2, respectively.

References

79. Kong, D.; Soon, C.S.; Chee, M.W. Reduced visual processing capacity in sleep deprived persons. *Neuroimage* **2011**, *55*, 629–634. https://doi.org/10.1016/j.neuroimage.2010.12.057.
80. Louca, M.; Short, M.A. The effect of one night's sleep deprivation on adolescent neurobehavioral performance. *Sleep* **2014**, *37*, 1799–1807. https://doi.org/10.5665/sleep.4174.
81. Brysbaert, M.; New, B. Moving beyond Kučera and Francis: A critical evaluation of current word frequency norms and the introduction of a new and improved word frequency measure for American English. *Behav. Res. Methods* **2009**, *41*, 977–990. https://doi.org/10.3758/BRM.41.4.977.

Figure S1

Object Detection Accuracy and Detection RTs: Control Originals and Replications

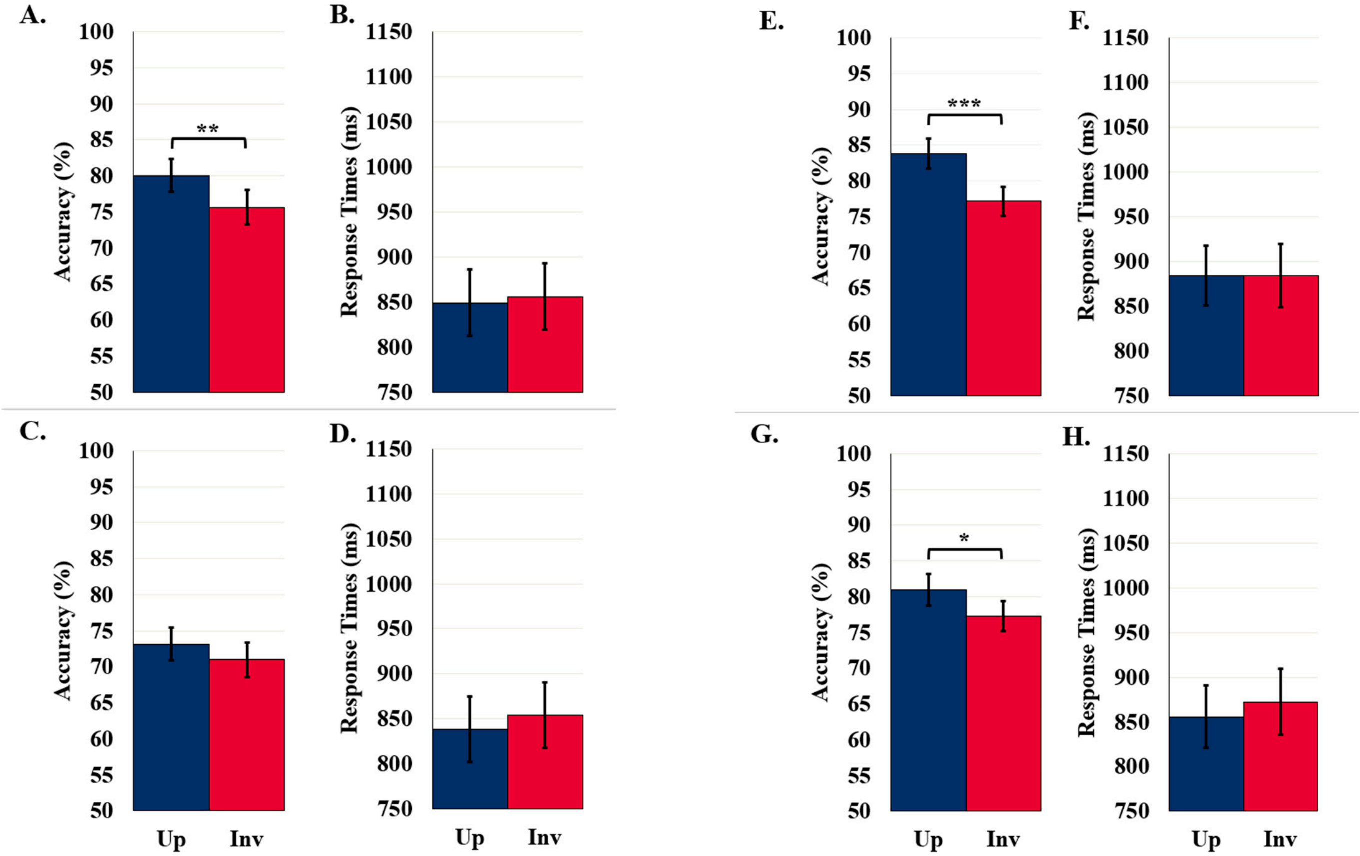

Results for individual control/labels-absent experiments. (**A–D**) 90-ms exposure durations [original: **A,B**; replication: **C,D**] and (**E–H**) 100-ms exposure durations [original: **E,F**; replication: **G,H**]; within each grouping, accuracy is on the left and response times are on the right. Up = upright displays; Inv = inverted displays. Error bars are standard error. *** indicates $p < 0.001$, ** indicates $p < 0.02$, and * indicates $p < 0.05$. The results of the original and replication experiments did not differ significantly for either accuracy or RTs: 90-ms accuracy $F(160) = 3.53$, $p = 0.065$; 90-ms RTs $F(157) = 0.01$, $p = 0.905$; 100-ms accuracy $F(157) = 0.24$, $p = 0.624$; 100-ms RTs $F(152) = 0.17$, $p = 0.685$. Therefore, the combined results are reported in the main text.

Figure S2

Object Detection Accuracy and Detection RTs: Study 1 Originals and Replications

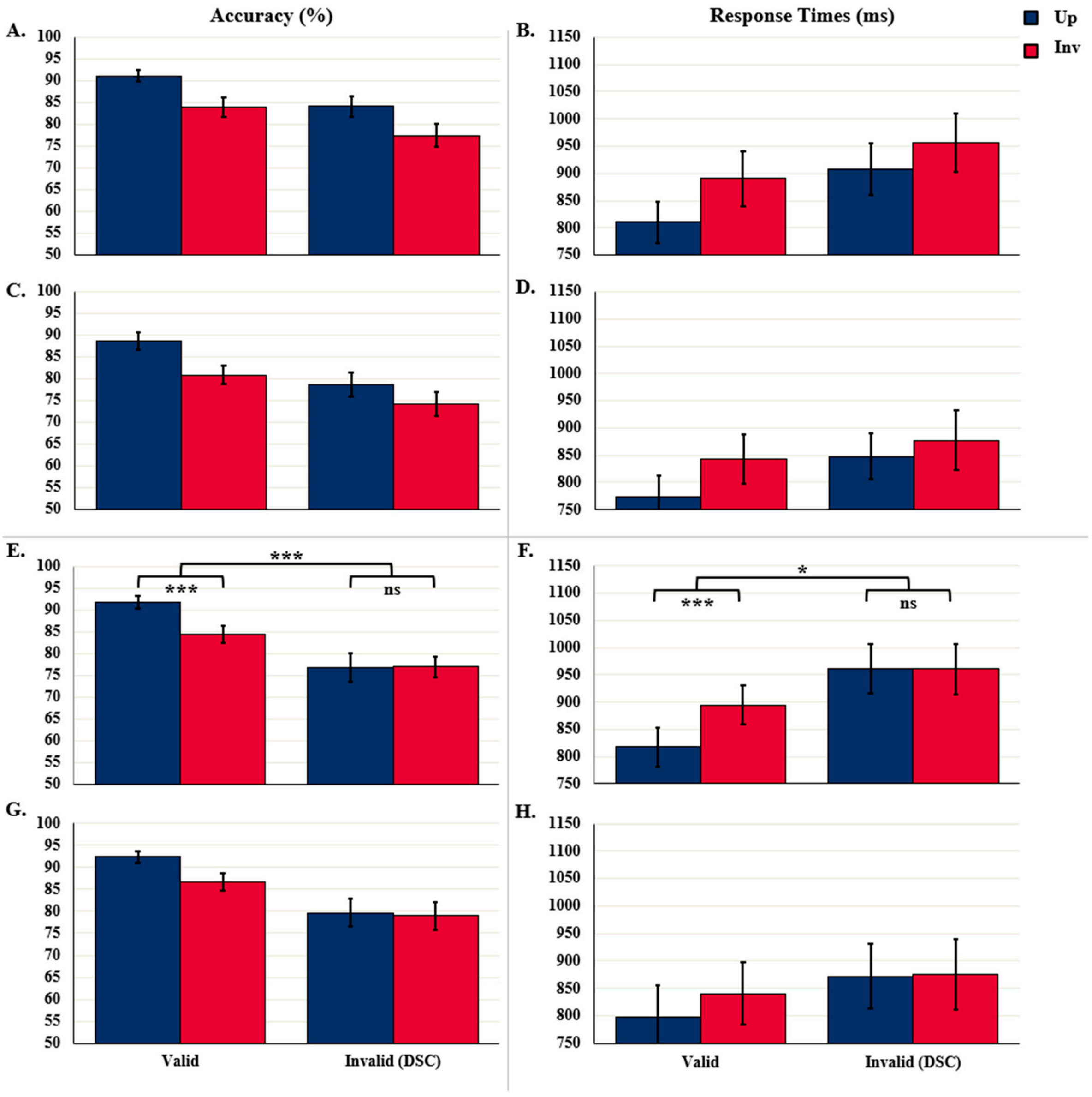

Results for individual Study 1 experiments. (**A–D**) 90-ms exposure durations [original: **A,B**; replication: **C,D**] and (**E–H**) 100-ms exposure durations [original: **E,F**; replication: **G,H**]. Accuracy is on the left and RTs are on the right. For accuracy, the main effects of orientation and label type were significant in all experiments, *p*s < 0.04 and *p*s < 0.001, respectively. For RTs, the main effects of orientation and label type were significant in all experiments, *p*s < 0.05 and *p*s < 0.02, respectively. Up = upright displays; Inv = inverted displays; Valid = a valid label preceded the bipartite display; Invalid (DSC) = an invalid label denoting a semantically unrelated object from a different superordinate-level category (natural vs. artificial) preceded the bipartite display. Error bars are standard error. *** indicates p < 0.008, * indicates p < 0.04, and ns indicates non-significance. The results of the original and replication experiments did not differ significantly for either accuracy or RTs: 90-ms accuracy $F(1,52) = 1.81$, $p = .184$; 90-ms RTs $F(1,49) = 0.80$, $p = .376$; 100-ms accuracy $F(1,56) = 0.61$, $p = .439$; 100-ms RTs $F(1,52) = 0.83$, $p = .365$. Therefore, the combined results are reported in the main text.

Figure S3

Object Detection Accuracy and Detection RTs: Study 2 Originals and Replications

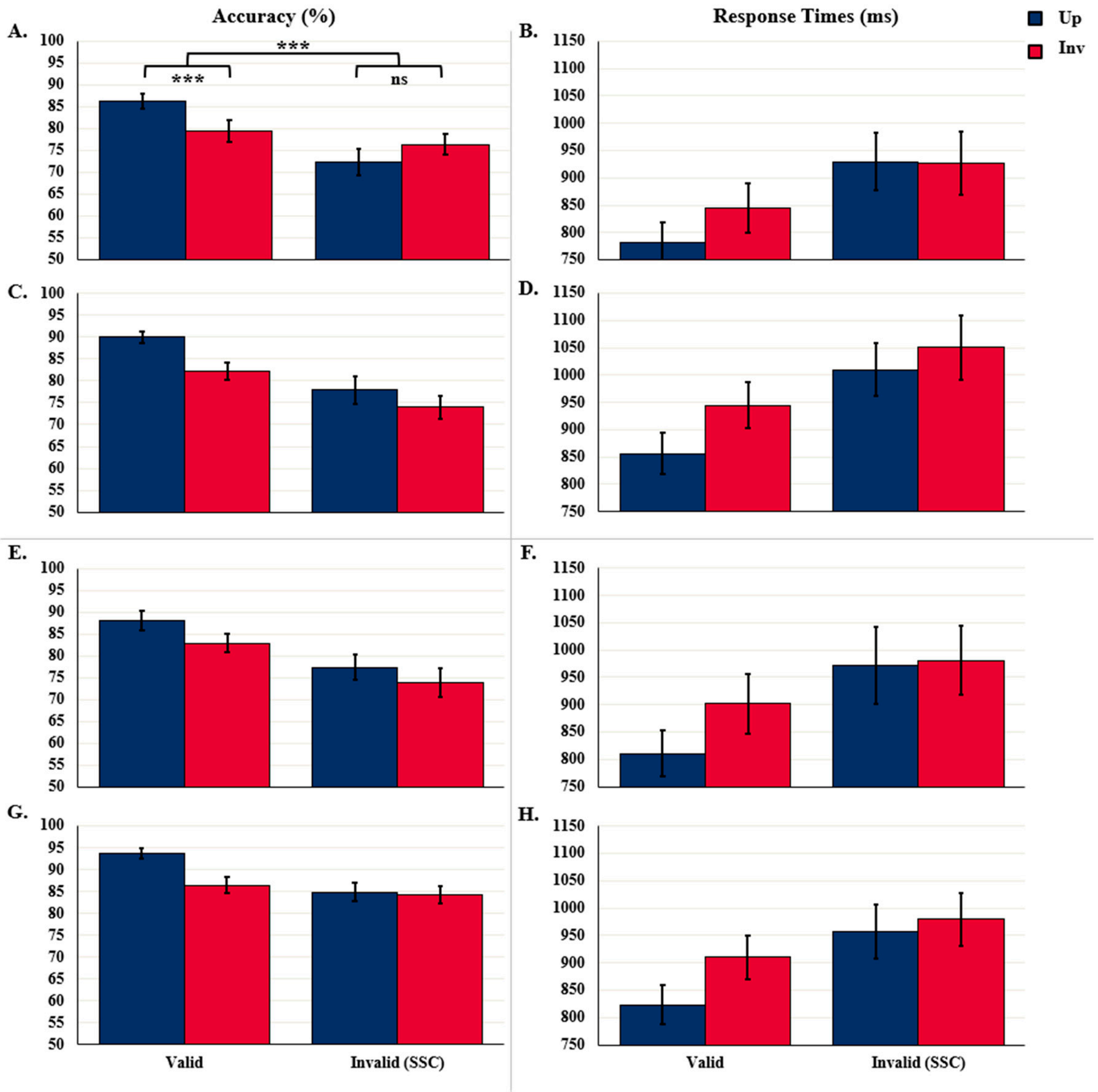

Results for individual Study 2 experiments. (**A–D**) 90-ms exposure durations [original: **A,B**; replication: **C,D**] and (**E–H**) 100-ms exposure durations [original: **E,F**; replication: **G,H**]. Accuracy is on the left and RTs are on the right. For accuracy, the main effects of orientation and label type were significant in all experiments except 90-ms original (**A**); significant effect *p*s < 0.02 and *p*s < 0.002, respectively. For RTs, the main effects of orientation and label type were significant in all experiments except 90-ms original (**B**); significant effect *p*s < 0.03 and *p*s < 0.003, respectively. Up = upright displays; Inv = inverted displays; Valid = a valid label preceded the bipartite display; Invalid (SSC) = an invalid label denoting a semantically unrelated object from the same superordinate-level category (natural vs. artificial) preceded the bipartite display. Error bars are standard error. *** indicates p < 0.005 and ns indicates non-significance. The results of the original and replication experiments did not differ significantly for 90-ms accuracy $F(1,54) = 0.93$, $p = .339$; 90-ms RTs $F(1,49) = 2.32$, $p = .134$; or 100-ms RTs $F(1,50) = 0.00$, $p = .985$. For experiments using 100-ms exposures, accuracy was significantly higher in the replication (87.4%) than the original (80.5%) experiments, $F(1,55) = 8.00$, $p = .007$, $\eta^2 = 0.13$ but experiment type did not significantly interact with any other factors, *p*s > .143. Therefore, the combined results are reported in the main text.
